# Metformin Use and Severe Dengue in Diabetic Adults

**DOI:** 10.1038/s41598-018-21612-6

**Published:** 2018-02-20

**Authors:** Htet Lin Htun, Tsin Wen Yeo, Clarence C. Tam, Junxiong Pang, Yee Sin Leo, David C. Lye

**Affiliations:** 1 Institute of Infectious Diseases and Epidemiology, Tan Tock Seng Hospital, Singapore, Singapore; 2 Lee Kong Chian School of Medicine, Nanyang Technological University, Singapore, Singapore; 3 Saw Swee Hock School of Public Health, National University of Singapore, Singapore, Singapore; 4 Department of Infectious Diseases Epidemiology, London School of Hygiene and Tropical Medicine, London, United Kingdom; 5 Yong Loo Lin School of Medicine, National University of Singapore, Singapore, Singapore

## Abstract

Diabetes mellitus is a risk factor for severe dengue in adults, but few studies have examined the association between metformin use and disease severity in dengue. In addition to its effect on glucose control, metformin has been associated with pleiotropic properties in preclinical studies. Using a cohort of laboratory-confirmed adult (≥21 years) dengue patients with diabetes mellitus admitted to Tan Tock Seng Hospital, we conducted a retrospective cohort study involving 131 (58.7%) metformin users and 92 (41.3%) non-users. Dengue severity was categorized as dengue hemorrhagic fever (DHF) or dengue shock syndrome (DSS) in World Health Organization (WHO) 1997 criteria and severe dengue (SD) in WHO 2009 criteria. Multivariable Poisson regression with robust error variance was used to estimate risk ratio (RR). Compared with non-use, metformin use was associated with a decreased risk of developing severe dengue (adjusted risk ratio [aRR] = 0.60, 95% confidence interval [CI]: 0.37–0.98, *P* = 0.04). Additionally, there was an inverse dose-response relationship (aRR = 0.69, 95% CI: 0.49–0.98, *P* = 0.04) with dengue severity as classified by WHO 2009 criteria. Use of metformin, however, was not associated with dengue severity based on WHO 1997 criteria; and no dose-response relationship was noted. Our results suggest metformin use could attenuate disease severity in dengue-infected diabetes mellitus individuals.

## Introduction

Dengue is the most common arthropod-borne viral infection, and the number of reported cases are increasing in the Western Pacific, the Americas and even Europe^1^. It is caused by four antigenically distinct serotypes of dengue viruses (DENV-1, DENV-2, DENV-3 and DENV-4) belonging to the *Flavivirus* family, all of which are transmitted by the *Aedes aegypti* and *Aedes albopictus* mosquitoes^2^. An estimated 390 million dengue infections occur worldwide each year, including 96 million with clinical symptoms. Asia is projected to have the highest burden, with 273 million infections annually^3^. Dengue infection manifests from asymptomatic infection to mild undifferentiated fever to a severe life-threatening condition. The World Health Organization (WHO) 1997 and the more recent WHO 2009 dengue classification criteria are the two most common classification criteria used to define dengue severity.

Against this background of increasing dengue incidence worldwide, the non-communicable disease burden is also growing. Among this group, diabetes mellitus (DM) is one of the most important and prevalent. According to the WHO, DM currently affects 347 million individuals worldwide, and it is predicted to be the seventh leading cause of death by 2030^4^. Of all the oral hypoglycemic agents (OHAs) to treat DM, metformin which belongs to the biguanide family is the most commonly prescribed and widely used agent. The blood glucose lowering action of metformin is achieved by suppressing gluconeogenesis^5^ and increasing peripheral tissue sensitivity to insulin^6,7^. In addition, metformin has also been demonstrated to have multiple pleiotropic actions including anti-inflammatory^8^, immunomodulatory^9^, anti-cancerous^10^, cardio-protective and vasculo-protective actions^11^ and limits the growth of micro-organisms^12–14^. Given dengue pathogenesis is a complex intertwined process involving the humoral and cellular immune systems, inflammatory cytokines and chemical mediators^15^, there may be a potential reduction in the risk of severe dengue manifestations among diabetic patients on metformin treatment because of immunomodulatory and anti-inflammatory properties.

Many observational studies have identified DM as an independent risk factor for severe dengue^16–19^. On the other hand, several observational studies in Asia and Europe have suggested a reduced risk of viral or bacterial infections and infection-related complications in metformin users^12,20,21^. To our knowledge, no studies have examined the role of metformin on dengue disease severity among diabetic patients independent of its effect on blood glucose.

We performed a retrospective cohort study among adult DM patients with acute dengue infection to evaluate the association between metformin use and risk of severe dengue, using both WHO 1997 and WHO 2009 dengue classification criteria.

## Methods

### Setting and data source

The study included patients admitted to the Department of Infectious Diseases, Tan Tock Seng Hospital (TTSH), the largest university teaching hospital in Singapore in dengue treatment. The source population was identified from three previous hospital-based dengue studies conducted in TTSH in 2004^22^, 2005–2008^23^ and 2012–2013. All of these studies included adult laboratory-confirmed dengue patients i.e., either positive dengue polymerase chain reaction (PCR)^24^ or non-structural protein 1 (NS1) antigen tests based on a single acute sample^25,26^. The databases of the three studies provided information on demographics, dengue laboratory test results, comorbidities and detailed clinical data for the present study.

### Study design and population

We conducted a hospital-based retrospective cohort study among adult (≥21 years) diabetic patients presenting with dengue. From the described source population, the study population was selected with the following eligibility criteria:

- Clinical manifestations of acute dengue infection according to WHO 1997 or 2009 guidelines with laboratory confirmation by either positive dengue PCR^24^ or NS1 antigen^25,26^ and
- Documented history of DM in hospital electronic medical records. Gestational diabetes without progression to DM was excluded.

### Exposure data

Participants were followed up from the date of presentation with dengue. The exposure of interest was current treatment with metformin assessed by tracking the most recent prescription from hospital medication prescription database records in 180 days prior to hospital presentation. Metformin usage was confirmed with medication reconciliation records performed by hospital pharmacists which listed all the medications patients were taking before hospitalization. Patients were classified as metformin users if they were on metformin treatment at the time of presentation and non-users otherwise. Metformin exposure was categorized as follows:

- *Users*: When there was a record for metformin use in the 180 days prior to hospital presentation, the prescription start date and duration of drug supply were collected to calculate the prescription end date. Patients were categorized as metformin users if the prescription end date was on or after the hospitalization date for dengue.
- *Non-users*: Patients with a metformin prescription ending before the hospital presentation date, or without any record of metformin use, were categorized as non-users.
- *Prescribed metformin doses*: Data on metformin dose was extracted for each user and was computed by multiplying the prescribed dose by daily prescription frequency.

### Outcome data

The study outcome, dengue severity was categorized as dengue hemorrhagic fever (DHF) and dengue shock syndrome (DSS) based on WHO 1997 criteria and severe dengue (SD) according to WHO 2009 criteria. Patients were retrospectively classified into the two WHO dengue criteria with available clinical, laboratory and radiological data in each study using the same criteria across all previous studies. Clinical variables during hospital stay were documented using a standardized dengue care path for all dengue patients and the data were extracted by medically trained research assistants from the first day of hospital presentation until discharge date for inpatients, and to the end of acute follow-up for outpatients.

#### WHO 1997 classification

Dengue fever (DF) was diagnosed when there was fever and two or more of: headache, eye pain, myalgia, arthralgia, rash, hemorrhagic manifestations or leucopenia. DHF was diagnosed when there was fever and all three of: hemorrhagic manifestations; thrombocytopenia <100 × 10^9^/L; and plasma leakage evidenced by pleural effusion or ascites or change in haematocrit ≥20% or hypoproteinaemia. To classify as DSS, there must be DHF as well as additional criteria including tachycardia with narrow pulse pressure lower than 20 mmHg or hypotension for age (systolic blood pressure <90 mmHg)^27^.

#### WHO 2009 classification

Probable dengue was diagnosed when there was fever and two or more of: nausea, vomiting, rash, aches and pains, leukopenia and presence of any warning signs which include abdominal pain or tenderness, persistent vomiting, clinical fluid accumulation, mucosal bleeding, lethargy or restlessness, liver enlargement >2 cm and increase in hematocrit concurrent with rapid decrease in platelet count. Severe dengue was defined as: (i) severe plasma leakage with respiratory distress or shock; (ii) severe bleeding defined as a minimum of WHO grade 2 bleeding scale including hematemesis, melena, hematochezia, hematuria, menorrhagia, hemoptysis or any other bleeding that required whole blood or packed red cell transfusion; (iii) severe organ involvement defined by either acute liver injury – aspartate transaminase (AST) and alanine transaminase (ALT) ≥1000 unit/L or acute kidney injury or encephalopathy or myocarditis^28^.

### Potential confounders

Covariates likely to be associated with dengue severity and likelihood of receiving metformin treatment were identified. These covariates were measured at the same time period as metformin exposure. Potential confounders included age^29^, gender^30^, ethnicity^31^, obesity^32,33^ as defined by body mass index (BMI) ≥30 kg/m^2^, smoking, and concurrent medications including other OHAs, statins, angiotensin-converting-enzyme inhibitors (ACEI) and angiotensin-receptor blockers (ARB)^34^. Dengue serotype is known to influence disease severity in Singapore^35^. However, patient-specific dengue serotype data for each subject was unavailable; thus the year of presentation was used as a surrogate index to estimate dominant circulating serotype in each epidemic year. Based on the Singapore communicable diseases surveillance data published by Ministry of Health Singapore, during the year of 2004 to 2006 and 2013, DENV-1 serotype predominated (61–82% of all circulating serotypes), and DENV-2 was the predominant serotype during 2007–2008 and 2012 (66–87%)^36^.

Comorbidities were measured by Charlson’s comorbidity index (CCI)^37^, and other comorbidities included hypertension, hyperlipidemia, allergy and past dengue^38^. Diabetes control was determined using glycated hemoglobin (HbA1c)^39^ measured in the 3 months prior to the dengue presentation date and categorized into ≤7.0% (optimal), 7.1–8.0% (suboptimal) and >8.0% (unacceptable). The diabetes complications severity index (DCSI)^40^, a validated 13-point clinical tool from seven categories of diabetes-related complications, was also included as a potential confounder. The decision to prescribe metformin depends on the renal function of patients. Therefore, serum creatinine >2.0 mg/dL and nephropathy on admission described in previous study^40^ were included. The diagnostic data to compute CCI and DCSI were drawn from the TTSH healthcare intelligence (TTSH-HI) system using the validated International Classification of Diseases Ninth Revision, Clinical Modification (ICD-9-CM) coding algorithms based on inpatient and outpatient encounters registered in TTSH-HI before the admission date. TTSH-HI is an integrated system with consolidated information from different sources to allow retrieval of structured variables such as demographic, clinical, laboratory and medication prescription data of each subject.

### Data collection

Microsoft Access database 2013 was used to collect, manage and collate the data. Data extraction was performed by two trained research assistants independently following standardized data collection procedures. Rule-based data validation was performed through the entire dataset to ensure data integrity and internal consistency. Additionally, 20% of the subjects were randomly selected for repeat data entry by another research assistant, and data discrepancies were resolved by independent source document verification by a senior medically-trained study team member. All subject identifiers were anonymized for analysis.

### Statistical methods

Characteristics of metformin users and non-users were described with frequencies and percentages, and median and interquartile range (IQR) for categorical and continuous variables respectively. Pearson’s chi-square test or Fisher’s exact test for categorical variables and Mann-Whitney U test for continuous variables were used for bivariate inference methods. We used Poisson regression with robust error variance^41^ to assess the association between metformin use and severe dengue manifestations, adjusting for potential confounding variables. In order to build the model, a forward stepwise variable selection approach was used to add the a *priori* defined clinically important variables including age, gender, ethnicity, CCI, DCSI, nephropathy, serum creatinine >2.0 mg/dL, HbA1c, year of presentation and variables with *P* < 0.20 between the metformin non-users and users. The best fit model, eventually, was compared against nested models evaluating with Akaike information criterion (AIC). Univariable and multivariable Poisson regression with robust error variance analyses provided crude and adjusted risk ratio (cRR and aRR) respectively with 95% confidence interval (CI). As a secondary analysis, the linear trend of dose-response relationship was studied and prescribed metformin doses were also categorized into no use, ≤1500 mg and >1500 mg based on the median prescribed dose.

All statistical analyses were conducted with Stata/SE14.0 (College Station, TX: StataCorp LP). Statistical significance was set at *P* < 0.05 and all reported *P* values were two-tailed.

### Ethics consideration and STROBE statement

This study was approved by Domain Specific Review Board of National Healthcare Group, Singapore (DSRB – 2015/01053) with waiver of informed consent from subjects. This study is reported in accordance with STROBE guidelines for cohort studies (see Supplementary Table S1).

### Data availability

All data supporting the findings of this study are available within the article and its Supplementary Information files, or are available from the corresponding author upon reasonable request.

## Results

**Figure 1:**
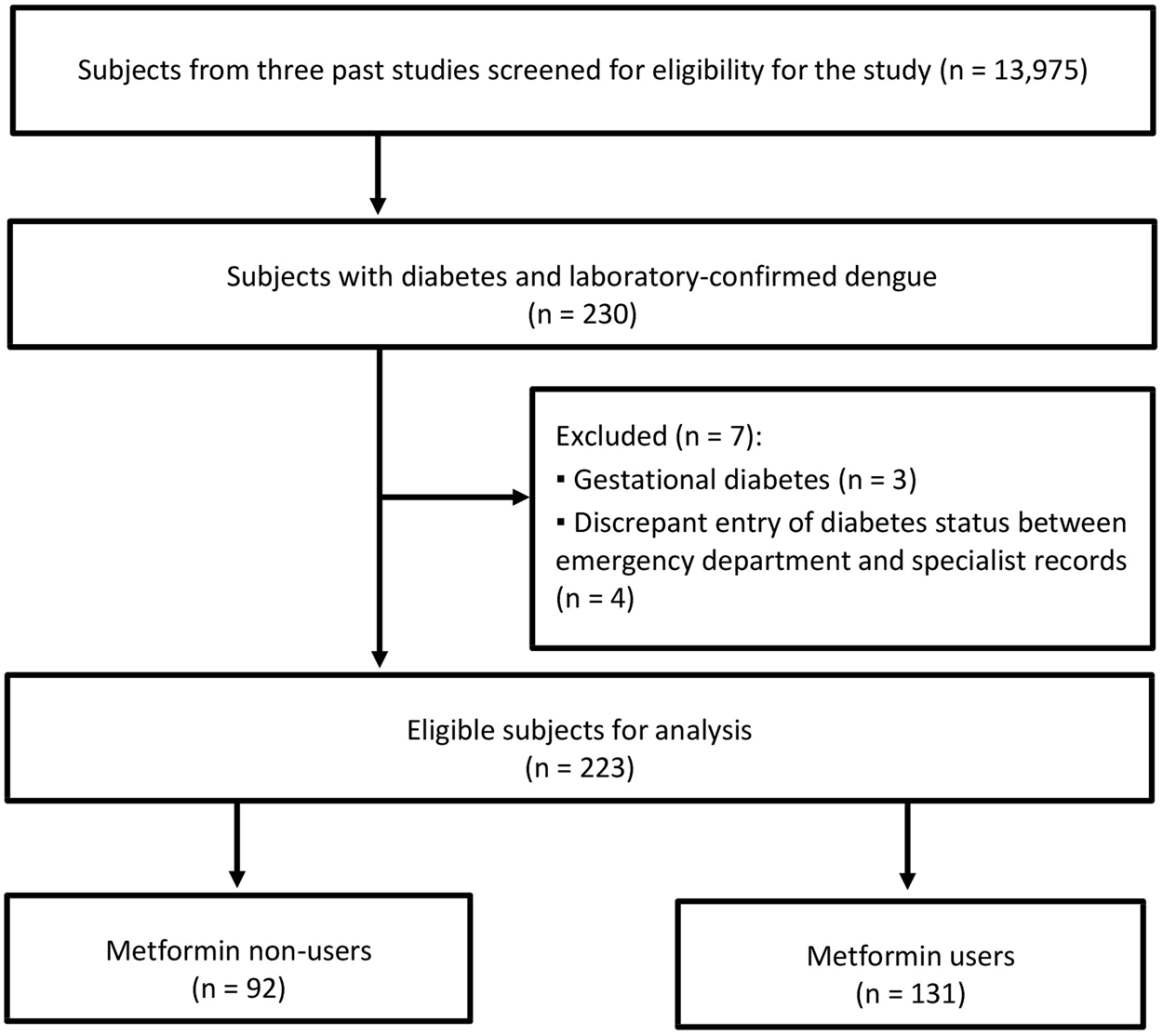
Flow diagram for selection of subjects.

From 2004 to 2008 and 2012–2013, we screened 13,975 potential subjects from the three past studies and identified 230 diabetic subjects with laboratory-confirmed acute dengue. Of these, 223 subjects met the eligibility criteria. Among eligible patients, 131 received metformin treatment (Fig. 1).

The median age was 58 (IQR: 49–69.5) and 59 (IQR: 50–69) years for metformin non-users and users respectively. There were no significant differences between metformin users and non-users in terms of gender (*P* = 0.88), ethnicity (*P* = 0.09), proportion of overweight and obese patients (BMI ≥25–29.99 and ≥30 respectively, *P* = 0.57), proportion of current smokers (*P* = 0.48), median CCI (*P* = 0.47), median DCSI (*P* = 0.60), nephropathy (*P* = 0.39), HbA1c category (*P* = 0.27), serum creatinine >2.0 mg/dL (*P* = 0.20) or predominant circulating serotype (*P* = 0.07) (Table 1). There was a statistically significant difference in the proportion of hyperlipidemia (*P* = 0.01) whereas no difference was observed for other comorbidities.

**Table 1:** Baseline demographic and clinical characteristics of metformin non-users and users; and users with standard prescribed metformin dose (≤1500 mg) and high prescribed metformin dose (>1500 mg).

**Variables**	**Non-users**	**Users**	**Users (n = 131)**		***P***_***1***_	***P***_***2***_
	(n = 92)	(n = 131)	≤1500 mg (n = 82)	>1500 mg (n = 49)		
**Demographic**						
Age - years					0.84^†^	0.70^‡^
Median (IQR)	58 (49–69.5)	59 (50–69)	60 (50–70)	58 (50–66)		
Range	25–93	29–85	35–85	29–85		
Gender, n (%)					0.88	0.99
Male	44 (47.8)	64 (48.9)	40 (48.8)	24 (49.0)		
Female	48 (52.2)	67 (51.1)	42 (51.2)	25 (51.0)		
Ethnicity, n (%)					0.09	0.12^∫^
Chinese	62 (67.4)	92 (70.2)	62 (75.6)	30 (61.2)		
Malay	13 (14.1)	7 (5.3)	3 (3.7)	4 (8.2)		
Indian	14 (15.2)	22 (16.8)	11 (13.4)	11 (22.4)		
Others	3 (3.3)	10 (7.6)	6 (7.3)	4 (8.2)		
**Health Risk Factor**						
Body mass index (BMI), n (%)					0.57	0.25
≤24.99	49 (53.3)	66 (50.4)	47 (57.3)	19 (38.8)		
≥25–29.99	28 (30.4)	48 (36.6)	26 (31.7)	22 (44.9)		
≥30	15 (16.3)	17 (13.0)	9 (11.0)	8 (16.3)		
Current smoker, n (%)	6 (6.5)	12 (9.2)	7 (8.5)	5 (10.2)	0.48	0.70^∫^
**Comorbidity**						
Charlson’s comorbidity index (CCI) [linear] Median (IQR)	1 (1–3)	1 (1–4)	1 (1–4)	1 (1–4)	0.47^†^	0.55^‡^
Charlson’s comorbidity index (CCI) [categorical], n (%)					0.26	0.46^∫^
1	52 (56.5)	71 (54.2)	42 (51.2)	29 (59.2)		
2	15 (16.3)	15 (11.4)	11 (13.4)	4 (8.2)		
3	7 (7.6)	6 (4.6)	3 (3.7)	3 (6.1)		
≥4	18 (19.6)	39 (29.8)	26 (31.7)	13 (26.5)		
Other comorbidities, n (%)						
Hypertension	61 (66.3)	99 (75.6)	59 (72.0)	40 (82.0)	0.13	0.16
Hyperlipidemia	62 (67.4)	107 (81.7)	62 (75.6)	45 (92.0)	0.01	<0.01
Allergy	17 (18.5)	35 (26.7)	23 (28.1)	12 (24.5)	0.15	0.32
Past dengue	2 (2.2)	7 (5.3)	5 (6.1)	2 (4.1)	0.31^∫^	0.41^∫^
**Complications**						
Diabetes complications severity index (DCSI) [linear] Median (IQR)	0 (0–2)	0 (0–2)	0 (0–2)	0 (0–2)	0.60^†^	0.86^‡^
Diabetes complications severity index (DCSI) [categorical], n (%)					0.95^∫^	0.92^∫^
0	56 (60.9)	73 (55.7)	44 (53.7)	29 (59.2)		
1	10 (10.9)	18 (13.8)	13 (15.9)	5 (10.2)		
2	9 (9.8)	16 (12.2)	12 (14.6)	4 (8.2)		
3	7 (7.6)	12 (9.2)	7 (8.5)	5 (10.2)		
4	4 (4.3)	5 (3.8)	3 (3.7)	2 (4.1)		
≥5	6 (6.5)	7 (5.3)	3 (3.7)	4 (8.2)		
Nephropathy, n (%)	18 (19.6)	32 (24.4)	21 (25.6)	11 (22.5)	0.39	0.63
**Laboratory values**						
Glycated hemoglobin (HbA1c), n (%)					0.27^∫^	0.03^∫^
≤7.0%	41 (44.6)	47 (35.9)	37 (45.1)	10 (20.4)		
7.1–8.0%	20 (21.7)	34 (26.0)	21 (25.6)	13 (26.5)		
>8.0%	25 (27.2)	46 (35.1)	22 (26.8)	24 (49.0)		
Missing	6 (6.5)	4 (3.0)	2 (2.4)	2 (4.1)		
Serum creatinine >2.0 mg/dL, n (%)	10 (10.9)	8 (6.1)	5 (6.1)	3 (6.1)	0.20	0.51^∫^
**Other concurrent medications**						
Sulfonylurea, n (%)	27 (29.3)	79 (60.3)	41 (50.0)	38 (77.6)	<0.01	<0.01
Insulin, n (%)	10 (10.9)	20 (15.3)	8 (9.8)	12 (24.5)	0.34	0.05
Other OHAs, n (%)	2 (2.2)	7 (5.3)	4 (4.9)	3 (6.1)	0.31^∫^	0.46^∫^
Statins, n (%)	27 (29.3)	98 (74.8)	57 (69.5)	41 (83.7)	<0.01	<0.01
ACE inhibitors, n (%)	12 (13.0)	49 (37.4)	23 (28.1)	26 (53.1)	<0.01	<0.01
Angiotensin-receptor blockers, n (%)	11 (12.0)	24 (18.3)	11 (13.4)	13 (26.5)	0.20	0.06
**WHO dengue severity**						
WHO 1997 dengue classification, n (%)						
Dengue hemorrhagic fever/dengue shock syndrome	28 (30.4)	39 (29.8)	23 (28.1)	16 (32.6)	0.92	0.85
▪ Hemorrhagic manifestations	41 (44.6)	54 (41.2)	35 (42.7)	19 (38.8)	0.62	0.80
▪ Plasma leakage	48 (52.2)	65 (49.6)	38 (46.3)	27 (55.1)	0.71	0.58
WHO 2009 dengue classification, n (%)						
Severe dengue	39 (42.4)	37 (28.2)	26 (31.7)	11 (22.4)	0.03	0.05
▪ Severe bleeding	19 (20.7)	12 (9.2)	8 (9.8)	4 (8.2)	0.02	0.05
▪ Severe plasma leakage	35 (38.0)	50 (38.2)	28 (34.2)	22 (44.9)	0.99	0.47
▪ Severe organ impairment	19 (20.7)	19 (14.5)	13 (15.8)	6 (12.2)	0.23	0.42
**Epidemic year**						
Year of presentation, n (%)					0.07	0.15
2004, 2005, 2006, 2013 (DENV-1)	64 (69.6)	105 (80.1)	64 (78.1)	41 (83.7)		
2007, 2008, 2012 (DENV-2)	28 (30.4)	26 (19.9)	18 (22.0)	8 (16.3)		

*P*_1_, statistical test between non-users and users; *P*_2_, statistical test between non-users, standard prescribed metformin dose (≤1500 mg) and high prescribed metformin dose (>1500 mg) users.

^†^Mann-Whitney U test.

^‡^Kruskal-Wallis test.

^∫^Fisher’s exact test.

Other OHAs include thaizolidinediones, alpha-glucosidase inhibitors, dipeptidyl peptidase-4 (DPP-4) inhibitors and meglitinides.

Metformin users were observed to have a lower proportion of severe dengue (42.4% in non-users *v*. 28.2% in users, *P* = 0.03). Amongst the three criteria of severe dengue in WHO 2009 classification, a significantly lower proportion of metformin users experienced severe bleeding compared with non-users (20.7% *v*. 9.2%, *P* = 0.02). Table 1 displayed the difference between non-users and metformin users with standard dose (≤1500 mg) and high dose (>1500 mg). There was a higher proportion of HbA1c >8.0% in high dose users and the difference was statistically significant (*P* = 0.03).

### Multivariable analysis based on WHO 2009 dengue classification

There were 37 (28.2%) severe dengue patients among metformin users and a significant inverse association was observed with metformin use and severe dengue on WHO 2009 classification. Compared with non-users, metformin users had a 33% reduction in a risk of severe dengue (cRR = 0.67, 95% CI: 0.46–0.96, *P* = 0.03) on univariable analysis. The risk reduction was 40% (aRR = 0.60, 95% CI: 0.37–0.98, *P* = 0.04) after controlling for age, gender, ethnicity, CCI, DCSI, nephropathy, hyperlipidemia, serum creatinine >2.0 mg/dL, HbA1c and concurrent medications including statins, ACEI, sulfonylurea and insulin, and year of presentation (Table 2).

**Table 2:** Risk ratio for association between metformin exposure and severe dengue manifestations based on WHO 2009 dengue criteria.

Exposure	No of patients	No (%) of severe dengue	Crude RR (95% CI)	*P* value	Adjusted RR^a^ (95% CI)	*P* value	Adjusted RR^b^ (95% CI)	*P* value
**Metformin use:**								
Non users	92	39 (42.4)	1.00		1.00		1.00	
Users	131	37 (28.2)	0.67 (0.46–0.96)	0.03	0.64 (0.45–0.93)	0.02	0.60 (0.37–0.98)	0.04
**Metformin daily doses:**								
Non users	92	39 (42.4)	1.00		1.00		1.00	
≤1500 mg	82	26 (31.7)	0.75 (0.50–1.11)	0.15	0.71 (0.47–1.05)	0.09	0.64 (0.39–1.04)	0.07
>1500 mg	49	11 (22.5)	0.53 (0.30–0.94)	0.03	0.53 (0.30–0.94)	0.03	0.50 (0.25–0.99)	0.05
Doses (linear)	131	37 (28.2)	0.73 (0.57–0.95)	0.02	0.72 (0.56–0.94)	0.02	0.69 (0.49–0.98)	0.04

^a^Adjusted for age, gender, ethnicity.

^b^Adjusted for age, gender, ethnicity, Charlson’s comorbidity index and diabetes complications severity index groups, nephropathy, hyperlipidemia, serum creatinine >2.0 mg/dL, HbA1c group, concurrent medications usage including statins, ACEI, sulfonylurea and insulin, and year of presentation.

On secondary analysis, there was an inverse dose-response relationship between metformin doses and severe dengue manifestations. Compared with non-users, the risk of severe dengue was 36% lower for patients taking ≤1500 mg (aRR = 0.64, 95% CI: 0.39–1.04, *P* = 0.07) and 50% lower for those taking >1500 mg (aRR = 0.50, 95% CI: 0.25–0.99, *P* = 0.05). In addition, there was evidence supporting an inverse linear trend in this association (aRR = 0.69, 95% CI: 0.49–0.98, *P* = 0.04) (Table 2).

### Multivariable analysis based on WHO 1997 dengue classification

There were 39 (29.8%) of DHF/DSS patients among metformin users and use of metformin had no statistically significant association with dengue severity based on WHO 1997 classification even after adjustment of potential confounders (aRR = 0.91, 95% CI: 0.55–1.52, *P* = 0.73). Likewise, no significant dose-response relationship was observed (aRR = 1.12, 95% CI: 0.79–1.57, *P* = 0.53) (Table 3).

**Table 3:** Risk ratio for association between metformin exposure and severe dengue manifestations based on WHO 1997 dengue criteria.

Exposure	No of patients	No (%) of DHF/DSS	Crude RR (95% CI)	*P* value	Adjusted RR^a^ (95% CI)	*P* value	Adjusted RR^b^ (95% CI)	*P* value
**Metformin use:**								
Non users	92	28 (30.4)	1.00		1.00		1.00	
Users	131	39 (29.8)	0.98 (0.65–1.47)	0.92	0.90 (0.60–1.35)	0.61	0.91 (0.55–1.52)	0.73
**Metformin daily doses:**								
Non users	92	28 (30.4)	1.00		1.00		1.00	
≤1500 mg	82	23 (28.1)	0.92 (0.58–1.47)	0.73	0.82 (0.52–1.31)	0.41	0.82 (0.47–1.42)	0.48
>1500 mg	49	16 (32.7)	1.07 (0.65–1.78)	0.79	1.04 (0.63–1.72)	0.88	1.29 (0.67–2.47)	0.45
Doses (linear)	131	39 (29.8)	1.02 (0.79–1.33)	0.86	0.99 (0.76–1.31)	0.86	1.12 (0.79–1.57)	0.53

^a^Adjusted for age, gender, ethnicity.

^b^Adjusted for age, gender, ethnicity, Charlson’s comorbidity index and diabetes complications severity index groups, nephropathy, hyperlipidemia, serum creatinine >2.0 mg/dL, HbA1c group, concurrent medications usage including statins, ACEI, sulfonylurea and insulin, and year of presentation.

## Discussion

This retrospective cohort study of adult diabetic patients with acute dengue infection indicated that patients on metformin treatment had a 33–40% lower risk of developing severe dengue based on WHO 2009 dengue criteria. Risk of severe dengue was also significantly reduced with increasing metformin dose intensity. Based on WHO 1997 dengue severity criteria, the association was not evident and neither was the dose-response relationship.

As the study suggested significant inverse association only for the analysis based on WHO 2009 classification, the relationship between metformin and severe dengue may be specific only to pathophysiology documented in the WHO 2009 dengue criteria. There are three severity markers in WHO 2009 dengue criteria – severe plasma leakage, severe bleeding and severe organ involvement. The pathogenesis of dengue virus infection leading to severe bleeding is complex but the major mechanisms involved can be summarized as platelet consumption, endothelial cell damage, imbalance profile of cytokines and chemical mediators, and suppression of hematopoiesis^15^. Investigations from *in-vitro* studies have found that metformin had direct vascular anti-inflammatory effect by attenuation of the inflammatory cytokine response^8^ while *in-vivo* studies have shown an effect of metformin on increasing the size of hematopoietic stem cell compartment and enhancing the quiescent state in hematopoietic stem cells and progenitor cells^42^. The effect of metformin on endothelial cell function has been well-studied and many clinical and pre-clinical studies suggest that metformin improves endothelial cell function in animal models^43,44^, and also among chronic diabetic subjects^45,46^. Furthermore, metformin exerts antioxidant effects on platelets^47^, strengthening and stabilizing the thrombocyte colonies^48^. Therefore, under a causal assumption, the most plausible hypothesis of a decrease risk of severe bleeding in metformin-exposed diabetic dengue individuals can be collectively explained by these pleiotropic actions of metformin, which could interact and inhibit the pathogenic mechanisms of dengue infection that lead to severe bleeding manifestations.

Our study has several strengths. Firstly, to our knowledge, this is the first observational study to explore the relationship between metformin exposure and dengue severity in adults. Secondly, severe dengue was analyzed with two different dengue severity classifications. Thirdly, finding of a dose-response relationship provides strong, biologically plausible support for the link between metformin use and reduced risk of developing severe dengue^49^. Fourthly, since the standardized dengue care path was used for clinical documentation in TTSH during the whole study period, the clinical and laboratory data for the dengue outcome severity was consistent with minimal outcome information bias. Lastly, evidence of significant reduction in dengue severity associated with metformin from this study gives insights to advance further studies.

The current study had several limitations with the first being the retrospective nature with missing data being unavoidable. Other limitations included the lack of data to adjust for the effect of different dengue serotypes on disease severity as patient-specific dengue serotype data was not available for the whole study population. However, the year of presentation to hospital for each patient was used as a surrogate indicator to estimate the different circulating dengue serotypes in each epidemic year. Similarly, potential effect of prior serostatus on disease severity was not described as serostatus is not routinely investigated in clinical practice. Although compliance of metformin was unknown, metformin usage was ascertained by using not only medication prescription database system but also medication reconciliation records performed on the first day of hospital admission before clinical outcomes developed by the trained pharmacists; as such risk of metformin misclassification is low. Additionally, dengue severity classification was performed retrospectively with available clinical and laboratory data with a risk of outcome misclassification bias although it could be minimal as standardized dengue care path was used in the whole study period. Furthermore, as with any observational studies, there is potential residual confounding with respect to unknown confounding variables that were not collected for the present study. Finally, there is a risk of channelling bias i.e. prescription of metformin is dependent on patient’s characteristics such as age, gender, ethnicity, presence of comorbidities and diabetes related complications but was reduced by adjusting these potential confounding variables.

The study included only adult DM patients presenting with dengue to a single hospital. Dengue cases in this study may not be representative of dengue-infected individuals in the general population, because there may be mild or asymptomatic dengue patients with DM. Hence, the study results may not be generalizable to other populations of adult dengue patients with DM. Finally, the study was not sufficiently powered to examine the interactions and different effects metformin might exert on different subgroups of patients, for instance, based on gender, age, ethnic groups and DM complications.

Metformin is an essential drug to manage diabetic patients. This study provides preliminary evidence of a reduced risk of severe dengue among metformin users, and also on an inverse dose-response relationship. As dengue burden is rising^1^ and prevalence of diabetes mellitus is growing worldwide, the study findings are of major importance to the public health.

To date, no curative measure has been shown to be effective in treating dengue infection and supportive management is still the mainstay treatment option. Observational and interventional studies have also proved that prophylactic platelet transfusion is ineffective in preventing severe bleeding while adverse events are more commonly observed in transfused patients^23,50–52^. Our study suggested the potential protective effect of metformin on dengue severity particularly on severe bleeding. For future research, preclinical studies could be employed to evaluate the possible biological mechanism link between metformin use and risk of severe dengue.

In summary, our study findings showed that usage of metformin at presentation was associated with lower risk of developing severe dengue in hospitalized adult dengue diabetic patients, compared with non-users, in the absence of prior serostatus knowledge. In addition, a significant inverse dose-response relationship was noted. The results also suggest the difference in severe bleeding may be a factor in explaining the lower risk of severe dengue among metformin users.

## Electronic supplementary material

Supplementary Information
