# Supplementary material for: Metformin Use and Severe Dengue in Diabetic Adults
Source: Sci Rep. 2018 Feb 20;8:3344. doi: 10.1038/s41598-018-21612-6 (PMC5820327; doi:10.1038/s41598-018-21612-6)
Supplement: Supplementary file 1 — Supplementary Information [file 41598_2018_21612_MOESM1_ESM.doc]

**Metformin use and severe dengue in diabetic adults**

**Htet Lin Htun^1^, Tsin Wen Yeo^1,2^, Clarence C Tam^3,4^, Junxiong Pang^3^, Yee Sin Leo^1,2,3,5^, and David C Lye^1,2,5^**

^1^ Institute of Infectious Diseases and Epidemiology, Tan Tock Seng Hospital, Singapore

^2^ Lee Kong Chian School of Medicine, Nanyang Technological University, Singapore

^3^ Saw Swee Hock School of Public Health, National University of Singapore, Singapore

^4^ Department of Infectious Diseases Epidemiology, London School of Hygiene and Tropical Medicine, London, United Kingdom

^5^ Yong Loo Lin School of Medicine, National University of Singapore, Singapore

Supplementary Table S1: STROBE Statement—Checklist of items that should be included in reports of ***cohort studies***

|  | Item No | Recommendation |
| --- | --- | --- |
| **Title and abstract** | 1 | (*a*) Indicate the study’s design with a commonly used term in the title or the abstract Done |
|  |  | (*b*) Provide in the abstract an informative and balanced summary of what was done and what was found  Done |
| Introduction | | |
| Background/rationale | 2 | Explain the scientific background and rationale for the investigation being reported  Done |
| Objectives | 3 | State specific objectives, including any prespecified hypotheses  Done |
| Methods | | |
| Study design | 4 | Present key elements of study design early in the paper  Done |
| Setting | 5 | Describe the setting, locations, and relevant dates, including periods of recruitment, exposure, follow-up, and data collection  Done |
| Participants | 6 | (*a*) Give the eligibility criteria, and the sources and methods of selection of participants. Describe methods of follow-up  Done |
|  |  | (*b*) For matched studies, give matching criteria and number of exposed and unexposed – NA |
| Variables | 7 | Clearly define all outcomes, exposures, predictors, potential confounders, and effect modifiers. Give diagnostic criteria, if applicable  Done |
| Data sources/ measurement | 8* | For each variable of interest, give sources of data and details of methods of assessment (measurement). Describe comparability of assessment methods if there is more than one group  Done |
| Bias | 9 | Describe any efforts to address potential sources of bias  Done |
| Study size | 10 | Explain how the study size was arrived at – Not reported |
| Quantitative variables | 11 | Explain how quantitative variables were handled in the analyses. If applicable, describe which groupings were chosen and why  Done |
| Statistical methods | 12 | (*a*) Describe all statistical methods, including those used to control for confounding  Done |
|  |  | (*b*) Describe any methods used to examine subgroups and interactions – NA |
|  |  | (*c*) Explain how missing data were addressed – done |
|  |  | (*d*) If applicable, explain how loss to follow-up was addressed – NA |
|  |  | (*e*) Describe any sensitivity analyses – NA |
| Results | | |
| Participants | 13* | (a) Report numbers of individuals at each stage of study—eg numbers potentially eligible, examined for eligibility, confirmed eligible, included in the study, completing follow-up, and analysed  Done |
|  |  | (b) Give reasons for non-participation at each stage  Done |
|  |  | (c) Consider use of a flow diagram  Done |
| Descriptive data | 14* | (a) Give characteristics of study participants (eg demographic, clinical, social) and information on exposures and potential confounders  Done |
|  |  | (b) Indicate number of participants with missing data for each variable of interest  Done |
|  |  | (c) Summarise follow-up time (eg, average and total amount) – NA |
| Outcome data | 15* | Report numbers of outcome events or summary measures over time  Done |
| Main results | 16 | (*a*) Give unadjusted estimates and, if applicable, confounder-adjusted estimates and their precision (eg, 95% confidence interval). Make clear which confounders were adjusted for and why they were included  Done |
|  |  | (*b*) Report category boundaries when continuous variables were categorized  Done |
|  |  | (*c*) If relevant, consider translating estimates of relative risk into absolute risk for a meaningful time period – Not relevant |
| Other analyses | 17 | Report other analyses done—eg analyses of subgroups and interactions, and sensitivity analyses – Not reported |
| Discussion | | |
| Key results | 18 | Summarise key results with reference to study objectives  Done |
| Limitations | 19 | Discuss limitations of the study, taking into account sources of potential bias or imprecision. Discuss both direction and magnitude of any potential bias  Done |
| Interpretation | 20 | Give a cautious overall interpretation of results considering objectives, limitations, multiplicity of analyses, results from similar studies, and other relevant evidence  Done |
| Generalisability | 21 | Discuss the generalisability (external validity) of the study results  Done |
| Other information | | |
| Funding | 22 | Give the source of funding and the role of the funders for the present study and, if applicable, for the original study on which the present article is based – NA |

*Give information separately for exposed and unexposed groups.
